# A Simple Monte Carlo Framework to Assess Suicide Risk in Adolescents: A Study at a High School in Colombia

**DOI:** 10.3390/ijerph17103674

**Published:** 2020-05-22

**Authors:** Elias David Nino-Ruiz, Ana Maria Trejos-Herrera, Maria Yaquelin Exposito-Concepcion, Marjorie Rodriguez-Giraldo, Randy Steven Consuegra-Ortega, Claudia Guevara-Novoa

**Affiliations:** 1 Applied Math and Computer Science Lab, Department of Computer Science, Universidad del Norte, Barranquilla 080001, Colombia; rconsuegra@uninorte.edu.co; 2 Department of Psychology, Universidad del Norte, Barranquilla 080001, Colombia; atrejos@uninorte.edu.co (A.M.T.-H.); marjoier@uninorte.edu.co (M.R.-G.); 3 Department of Nursing, Universidad del Norte, Barranquilla 080001, Colombia; mexposito@uninorte.edu.co (M.Y.E.-C.); cguevara@uninorte.edu.co (C.G.-N.)

**Keywords:** Monte Carlo, covariance estimation, suicide risk, psychological instrument, 21.60.Ka, 52.65.Pp

## Abstract

It is very common to perform statistical tests to obtain insights about populations based on samples. For instance, in the context of psychology, when a set of instruments are applied to individuals, psychologists typically look for an explanation of particular psychological constructs (variables), such as personality, intelligence, or emotional functioning. It is common to cross statistical information from the results of different psychological tests to measure certain variables or to confirm prior beliefs. Here, we estimate the Joint Probability Density Function of suicide-related vulnerability and protective factors to assess suicide risk in adolescents. A Markov Chain Monte Carlo Method is employed to move away from the typical Gaussian assumption on data. This allows us to estimate probabilities of the development of suicidal ideation based on samples (which form a Markov chain). We employ our proposed statistical method at a high school in Colombia. The results reveal that adolescents can develop suicidal ideation as a consequence of the following factors, together with their corresponding probabilities: poor school performance 52%, low academic expectations 27%, school integration problems 68%, risky eating behaviors (binge-purge) 42%, risky eating behaviors (compensatory measurements) 21%, risky eating habits (restriction) 22%, and low family functionality 16%.

## 1. Introduction

The World Health Organization (WHO) recognizes suicide as a public health priority. Every year, close to 800,000 people take their own life, and there are many more people who attempt suicide. These series of events take place throughout people’s lifespans. Suicide was the second leading cause of decease in young people (age range between 18 and 29 years old) in 2016 [1]. Besides this, the WHO pointed out that 78% of worldwide cases were from low- and middle-income countries [2].

Suicidal behavior is a set of complex events, which can affect people of any age or condition; when it manifests as consumed suicide, the devastating effects impact on individuals, families, and societies: the effects are long-lasting. In [3], they explain that suicidal behavior is a sequence of events that are known as the suicidal process; this occurs progressively in most of the cases. Typically, it starts with suicidal thoughts/ideas that are followed by suicide plans, which can potentially lead to one or multiple suicide attempts. These events progressively increase in lethality until they take their life.

According to the National Institute of Legal Medicine and Forensic Sciences of Colombia [4,5,6], from 2009 to 2018, there were a total number of 20,832 suicides, with an average of 2083 cases per year and a rate of 5222 per 100,000 inhabitants older than five years. In Colombia, a significant percentage occurs in the young population (43.36%); this is between 18 and 29 years old, whose majority are men (82.34%). The progressive increase in suicidal behavior (10.53% of the total cases) in children and adolescents (between 5 and 17 years old) is all the more worrying [7].

Despite the fact that research in suicide prevention has proven to be useful [8], it has been constrained by factors such as short observation periods of potentially suicidal individuals, and high variability (uncertainty) in its main causes (e.g., sex, age, socio-demographic variables) [9]. The suicidal ideation is a set of thoughts that express a desire or intent to die, or other suicidal psychological experiences, such as the fantasy or foreshadowing of death itself [10,11].

Suicidal ideation is a complex phenomenon that involves personal, family, and school factors. The specialized literature has shown that there is a strong relationship between family functioning and suicidal ideation [12,13,14]. These studies suggest that high levels of suicidal ideation in adolescents are related to low perceptions of social support, less communication, and strong conflicts with parents and relatives. Likewise, with regard to variables such as school adjustment, strong negative correlations have been found with suicidal ideation [14,15,16]. This implies that adolescents with low school recognition and high levels of school victimization are likely to be more prone to suicidal ideation. Recent studies have obtained significant relationships between variables associated with eating disorders and suicidal ideation [17,18]. However, other studies suggest that there is no clear relation between high body mass index and suicidal ideation [19]. It is well-known that patients with eating disorders can have suicidal thoughts and self-injurious behaviors, but this association has not been formally defined [20].

Most of the studies that have been performed in this area rely on a frequentist analysis: they rely on the information brought by samples such as ANCOVA [21,22,23]. Moreover, there are studies that use a Bayesian framework on the estimation of spatial disparity in the risk of suicidal ideation [24] or by employing Gibbs sampling [25]. However, Gibbs sampling relies on probability distributions for which we know how to sample. This forces researchers to make assumptions on the data they are dealing with. We think that, by employing Monte Carlo sampling, there is an opportunity to estimate correlations between scores of scales/sub-scales from psychological instruments to assess suicide risk in populations and to explain its relationship with other psychological constructs. Besides, no assumptions are actually needed on the data to perform such analysis. This paper is organized as follows: in Section 2, psychological instruments, as well as statistical concepts, are discussed. Section 2.2 proposes a Monte Carlo framework to estimate Joint Probability Density Functions of psychological variables. In Section 3.1, we employ our proposed framework at a high school in Colombia. Section 5 states the conclusions of this research.

## 2. Materials and Methods

In this Section, we briefly discuss topics related to the derivation of our framework: we explain the psychological instruments to be employed, and furthermore, some statistical concepts are discussed in light of the psychological context.

### 2.1. Population and Psychological Instruments to Assess Suicide Risks

The population consisted of 775 students from the Simón Bolívar Tourist Educational Institution of Puerto Colombia from 6th to 11th grade, considering the literature reports the highest incidence of suicidal ideation in people over 12 years of age.

A random sample was selected and stratified by sex and grade level. The sample was made up of 413 adolescents (52.5% men and 47.5% women). Their ages ranged from 10 to 18 years. The average and standard deviation was 12.72 and 1.54, respectively, at the time of evaluation (men, 13.51 and 1.62, and women 12.72 and 1.54), which allowed the study groups to be relatively similar in size. Most adolescents (85.1%) lived in urban areas, while 55% professed the Catholic religion.

Parental and adolescent consent was requested. In each classroom, the questionnaires were given to the participants at agreed times with the school authorities. The questionnaires were all self-administered. The instruments were applied in the educational institution, at agreed times with the corresponding authorities. We avoided the effect of fatigue (and the possibility of false responses) by administrating the instrument at two different times with an interval of two days.

A pilot study was carried out to assess the understanding of the instruments in the context of the study, taking a sample that had similar characteristics to the population to be studied.

The inclusion criterion was as follows: adolescents enrolled in the Simón Bolívar Tourist Educational Institution at Puerto Colombia, who are in 6th to 11th grade, gave their consent, and also had a consent form signed by their parents or legal guardians.

We were able to estimate relationships between the discussed psychological variables by employing well-known instruments, some of them detailed below—these were employed by our Monte Carlo framework:

- The Positive and Negative Suicide Ideation (PANSI) inventory was proposed in [26]. This instrument was adapted to the Colombian context by Villalobos-Galvis in [27], and it is a self-reported measurement which consists of two sub-scales that evaluate suicide-related vulnerability and protective factors. This is a 14-item inventory that consists of two sub-scales: the Negative Suicide Ideation (PANSI-NSI) subscale (8 items), and the Positive Ideation (PANSI-PI) subscale (6 items). The total possible scores on the sub-scales PANSI-NSI and PANSI-PI ranged from 8 to 40 and 6 to 30, respectively. High scores on the PANSI-NSI and low scores on the PANSI-PI reflect high risks of suicidal behavior. Each item in PANSI is rated on a five-point Likert scale (1 = none of the time to 5 = most of the time).
- Family Adaptability, Partnership, Growth, Affection, and Resolve (APGAR) [28] is a scale that can be employed to assess family function. It is a five-item questionnaire (each item rated on a three-point scale) that measures five psychological constructs: Adaptability, Partnership, Growth, Affection, and Resolve. The patient can check one of three choices, which are scored as follows: “Almost always” (2 points), “Some of the time” (1 point), or “Hardly ever” (0 points). In this instrument, the total score can range from 0 to 10; individuals with high scores imply good family functionalities. Total scores ranging from 0 to 3 indicate a severely dysfunctional family; scores between 4 and 6, moderate dysfunction; total scores between 7 and 10, mild dysfunction; and other scores are considered functional.
- The Brief Scale of School Adjustment (BSSA-10) [29] is a 10-item scale, whose response format has six options, ranging from 1 (completely disagree) to 6 (completely agree). Five items of the scale are written in an inverse sense (items 6–10). The scores can range from 10 to 60. Individuals with high scores imply good school adjustment, such as good relationships with classmates and instructors, responding well to school activities (i.e., classroom and take-home assignments), motivation to attend school, and positive and integrated self-concepts as students. It has three sub-scales, which are: Scholarly performance (SP), with three items (1, 2, 5); Academic Expectations (AE), which has two items (3, 4); and Integration Problems (IP) with five items (6, 7, 8, 9, and 10).
- The Brief Questionnaire of Risk Eating Behaviors (BQREB) [30] was developed based on the DSM-IV diagnostics criteria. This instrument consists of 10 questions. These questions target different aspects of individuals: weight gain, binge eating, lack of control, restrictive eating behaviors (i.e., diets, fasts, and excessive exercise), and purgative conducts (i.e., self-induced vomiting, and the use of laxatives). The questions target individual behaviors based on three months prior to the application of the instrument. The BQREB is a 10-item scale; each response has four options, which ranges from 1 (i.e., almost never) to 4 (i.e., very frequently).
  The BQREB, as stated before, has a three-factor structure, which are: Binge-purge (BP), made up of four items; Compensatory measures (CM), consisting of three reagents; and Restriction (R), which includes the remaining three items.

### 2.2. Proposed Method: A Monte Carlo Based Methods to Assess Suicide Risk

Markov Chain Monte Carlo (MCMC) methods are powerful statistical tools that allowed us to estimate samples from probability density functions, for which sampling is not trivial (or basically, unknown) [31]. The idea behind these methods is to form a chain (of samples) which converge to high-probability zones of some target distributions. Samples are proposed by well-known probability functions (i.e., Normal distribution), and the Metropolis Hasting criterion is applied to accept or to reject candidates. We employed MCMC to draw samples from the Joint Probability Density Functions (PDF) π(x) of PANSI dimensions (factors) with those from BSSA-10, BQREB, and APGAR. Thus, we estimated the Joint PDFs of the following pairs of dimensions (factors): BSSA-10 SP-PANSI PNI, BSSA-10 SP-PANSI PSI, BSSA-10 AE-PANSI PNI, BSSA-10 AE-PANSI PSI, BSSA-10 IP-PANSI PNI, BSSA-10 IP-PANSI PSI, BQREB BP-PANSI PNI, BQREB BP-PANSI PSI, BQREB CM-PANSI PNI, BQREB CM-PANSI PSI, BQREB R-PANSI PNI, BQREB R-PANSI PSI, APGAR-PANSI PNI, and APGAR-PANSI PSI. We implemented our proposed method by using MATLAB^®^ 2020a version [32]. For each pair of factors, we approximated their Joint PDF by employing the Kernel Smoothing Function Estimate *mvksdensity*. By using this function approximation, we used the scores of a random individual to start our MCMC chain. Candidates for distribution π(x) had the form:

(1) $$\begin{array}{c} x_{c}∼N\left(x_{k},\;I\right)\;, \end{array}$$

where $N\left(•,\;•\right)$ denotes a Normal distribution, $x_{k}$ is the chain member at iteration *k*, and I is the two-dimensional Identity matrix. We then let:

(2) $$\begin{array}{l} x_{k+1}=\left\{\begin{array}{ll} x_{c} & \;\mathrm{with}\;\mathrm{probability}\;\min\left(1,\;J\left(x_{c}\right)/J\left(x_{k}\right)\right)\\ x_{k} & \;\mathrm{otherwise} \end{array}\right.\;. \end{array}$$

The steps (1) and (2) are repeated over a pre-defined number of iterations *N*. As a result, we obtain a chain ${\left\{x_{k}\right\}}_{k=0}^{N}$ that (approximately) satisfies:

(3) $$\begin{array}{c} x_{k}∼\pi \left(x\right)\;, \end{array}$$

and even more, it converges to high-probability zones of π(x). Usually, one discards the first of the *M* elements from the chain, since these correspond to the burning steps of MCMC (samples with low probabilities). Note that our method does not make any assumption on the data—we approximated a Kernel distribution based on our actual data, and then by employing a MCMC method, we reached high-probability zones of such Kernel approximation. To the best of our knowledge, there is no method in the current literature that works in such a manner.

## 3. Results

### 3.1. Study at a Secondary School in Colombia

In this section, we applied our proposed method to study suicide risk at a high school in Puerto Colombia, Colombia. The sample was made up of 413 students (52.5% men and 47.5% women). Their ages ranged between 10 and 18 years, the average and the standard deviation being 12.72 ± and 1.54 at the time of evaluation (men, 13.51 ± and 1.62, and women 12.72 ± and 1.54). Most of the adolescents (85.1%) lived in urban areas, while 55% of individuals professed the Catholic religion. The objectives and study procedures, including informed consent, were evaluated and approved by the Ethics Committee of Universidad del Norte in Barranquilla, Colombia, Act Number: 180. The ethical aspects of research involving human beings outlined in Resolution # 008430 of 1993 by the Ministry of Health and Social Protection of Colombia and the Code of Ethics for Psychologists Law 1090 of 2006 (also known as Psychologist’s law in Colombia) were taken into consideration during the preparation of this study; these include professional secrecy, the right to decline or withdraw participation, informed consent, and return of results. Each participant signed an informed consent form, wherein the objectives, procedures, risks, benefits, voluntary nature, and confidentiality of the study were clearly outlined. The descriptive statistics of the characteristics of the participants are shown in the Table 1 and Table 2.

### 3.2. Test Validation

**Figure 1:**
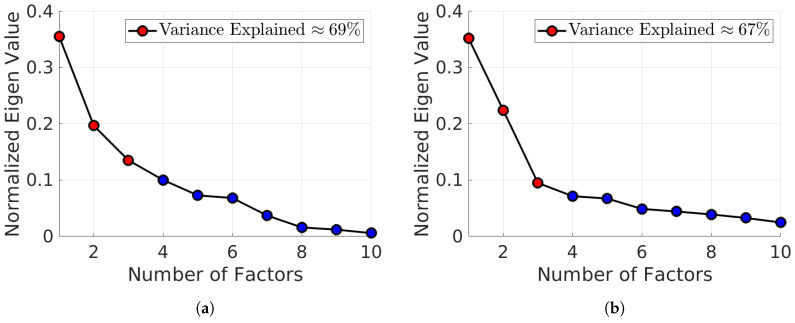
Percentage of variance explained as a function of the number of eigen values. For the BQREB and the BSSA-10, three eigen values explain more than 65% of the total variance. (**a**) BQREB. (**b**) BSSA-10.

**Figure 2:**
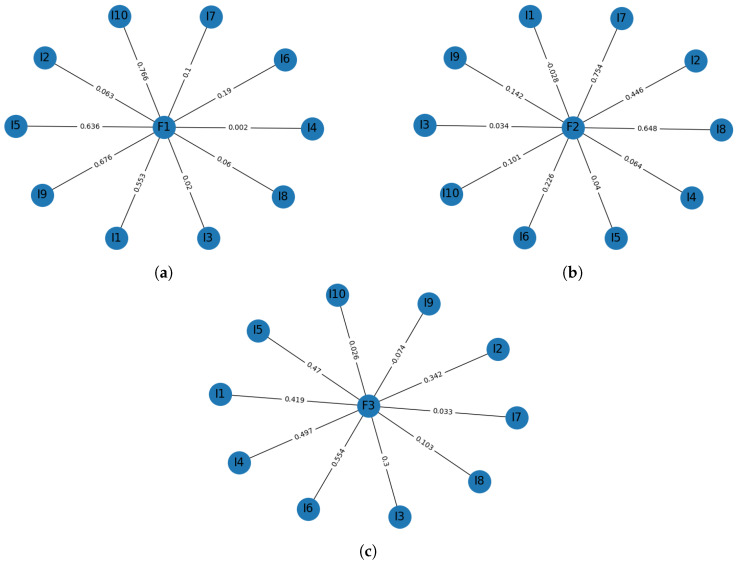
Factorial model for the BQREB instrument. (**a**) BQREB BP. (**b**) BQREB CM. (**c**) BQREB R.

**Figure 3:**
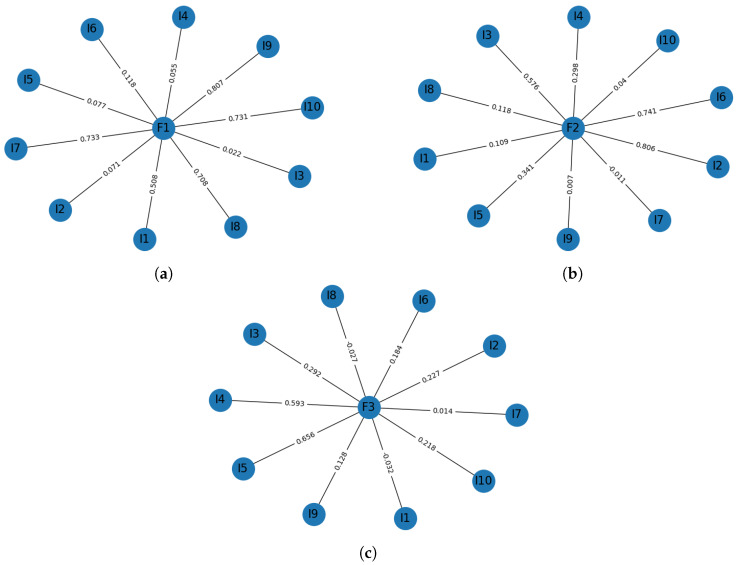
Factorial model for the BSSA-10 instrument. (**a**) BSSA-10 SP. (**b**) BSSA-10 AE. (**c**) BSSA-10 IP.

We performed a statistical validation for the BQREB and BSSA-10 tests. By using MATLAB®, the Kaise-Meyer-Olkin statistic (KMO) test for the sampling adequacy of BQREB was estimated. We found that our sample was well-suited to apply factor analysis (KMO ≥ 0.7269, yielding a degree of common variance middling), and applying Bartlett’s test to the correlation matrix, we were able to confirm our results ($\chi_{18}^{2}=58.1249,p<4.0960e\times 10{}^{-6}$). Using the Kaiser criterion [33], we found that the test had a three-factor structure. Overall, this factorial model explained 69% of the total variance, as shown in Figure 1a. Figure 2 depicts the structure of this model. We split the factors for ease of reading. Following these steps, based on the KMO for the BSSA-10 test, we found that our sample was well-suited to apply factor analysis (KMO ≥ 0.7269, yielding a degree of common variance middling) and we also confirmed this result ($\chi_{18}^{2}=84.0588,p<1.6545e-10$). Using the Kaiser criterion, we found that the test had a three-factor structure, as shown in Figure 2. Overall, this factorial model explained 67% of the total variance, as shown in Figure 1b. Figure 3 depicts the structure of this model.

### 3.3. Proposed Method Results

We start by analyzing the correlations of our samples; these ones have been reported in Table 3 for the compared instruments. We observe that:

1. We found a negative correlation [34] between APGAR and PANSI PSI/PANSI PNI, which implies that adolescents with high levels of suicidal ideation can be associated with a low perception of social support, and less communication and a high level of conflict with their parents and relatives;
2. We could see a negative correlation with BSSA SP and PANSI PSI/PANSI PNI. This suggests that adolescents with high levels of suicidal ideation can be related to low levels of school adjustment. Furthermore, they can have relationship problems with classmates and instructors, make little effort on school assignments (i.e., homework and class work), poor academic motivation, have poor self-concept as a student, and low expectations of pursuing high education levels;
3. The values obtained by the BQREB in the correlation analysis with the suicide risk scales also provide important information in agreement with the data reported in the literature on the comorbidity of eating disorders with depression and mortality associated with suicide [35]. It is worth mentioning that there is a greater number of investigations in the general population or adult clinic in comparison with the adolescent population about the association between suicidal ideation, self-injurious behavior and eating disorders, though this relationship is complex and has not yet been resolved [36].

**Figure 4:**
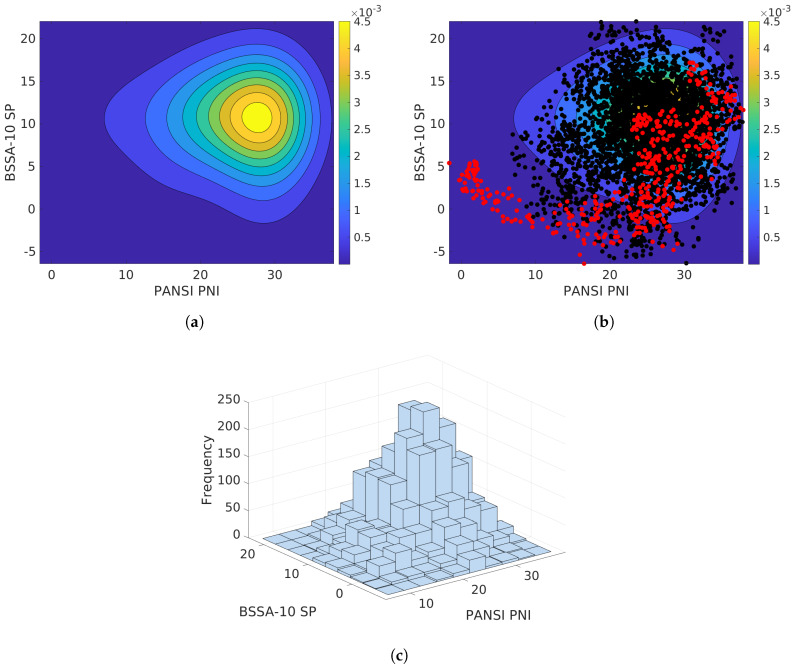
Correlations of scales/sub-scales for the employed instruments during studies. (**a**) Estimated Joint PDF. (**b**) Markov Chain. (**c**) Samples from the Joint PDF.

To move away from Gaussian assumptions on data and to estimate probabilities of potential psychological constructs which can detonate suicidal behaviors on adolescents, we proceed as follows (Section 2.2): for each scale/subscale in BQREB, APGAR, and BSSA-10, we estimate their Joint PDF with factors from the PANSI instrument. As we mentioned before, the estimation process is performed by using a Kernel smoothing estimation. In Figure 4a, we can see an example of a Joint PDF estimation for the BSSA-10 SP and the PANSI NPI scores. As can be seen, the approximated Joint PDF is non-Gaussian. We then proceed to employ a MCMC to draw samples from the Joint PDF. Figure 4b shows the chain formed by the MCMC method. Red dots denote samples which are discarded, given their low probability (burning steps), where black dots stand for points which are accepted as samples from the Joint PDF. We employed a total number of 20,000 samples. In Figure 4c, we show a histogram with samples from the Joint PDF. We do this for all PANSI dimensions (factors) with those from BSSA-10, BQREB, and APGAR.

By using the estimated samples from each Joint PDF, we evenly split their range (intervals) of possible scores for the different dimensions. By using the samples, we estimate the joint probabilities of PANSI dimensions vs. those of BSSA-10, BQREB, and APGAR. The probabilities are reported in the Table 4.

Table 4 shows the probability distribution values to obtain a certain score in a test, given the results for the PANSI, in our context. Higher values of probability mean that it is highly likely to obtain a value in that range, conditioned on the results of the PANSI-NSI. We can observe that most of the higher probability values lie in the mid-intervals, such as [20,29] for PANSI-PNI. This could be correlated to the socio-demographic context of the sample. Furthermore, we could estimate missing values on data to give a more accurate diagnosis interpretation. Note that the risk of generating suicidal behaviors, given low results from the subscales, PANSI-PNI and BSSA-10 SP, is up to 52%. In the same sense, given low results from BSSA-10 IP and PANSI PNI, we can estimate up to 68% of the risk of suicidal behaviors. To be concise:

- Adolescents with poor school performance have up to a 52% probability of developing a risk of suicidal ideation;
- Adolescents with low academic expectations have up to a 27% probability of developing a risk of suicidal ideation;
- Adolescents with school integration problems have up to a 68% probability of developing a risk of suicidal ideation;
- Adolescents with risky eating behaviors (binge-purge) have up to a 42% chance of developing a risk of suicidal ideation;
- Adolescents with risky eating behaviors (compensatory measurements) have up to a 21% probability of developing a risk of suicidal ideation;
- Adolescents with risky eating habits (restriction) have up to a 22% probability of developing a risk of suicidal ideation;
- Adolescents with low family functionality have up to a 16% probability of developing risk of suicidal ideation.

## 4. Discussion

Typically, a Joint PDF of scales from different instruments is non-Gaussian. This happens as a consequence of the ceiling effect, which can be observed in multiple sub-scales’ instruments. Hence, the proposed method can be exploited in such contexts to estimate Joint PDF, and even more, the computation of samples from these probability densities.

According to the WHO, adolescent suicide is associated with the changes that occur in this stage of life; however, there are also other adolescent problems related to suicide, such as behavioral disorders, bullying, sexual abuse, child abuse, eating disorders, anxiety, depression, risky behaviors, and alcoholism [37].

There is a lack of clarification and integration of the different models to more clearly determine the relevant conditions and factors in the suicide process, and there are probably not the same causes in all individuals, but rather, they are particular to the history of each person [38]. Therefore, an interdisciplinary and intersectoral approach is necessary for the study of this phenomenon.

## 5. Conclusions

In this study, we proposed an efficient and practical Monte Carlo [39] framework to assess suicide risk. The proposed method employed a Markov Chain Monte Carlo method to estimate Joint Probability Density Functions (PDF) of test scores from psychological instruments. The Joint PDFs allowed us to get insights about the potential cause/effect of any two variables, and even more, to provide probabilities. We applied our framework to individuals of a public school in Puerto Colombia, Colombia. The results revealed that adolescents with high levels of suicidal ideation are related to the low perception of social support, and less communication and conflicts with their parents. Moreover, adolescents with high levels of suicidal ideation were found to be associated with low school adjustment—for instance, they can have problems with classmates and instructors, make little effort in class assignments, have poor academic motivation, and poor self-concept as students. Besides this, adolescents can develop suicidal ideation as a consequence of the following factors, together with their probabilities: poor school performance 52%, low academic expectations 27%, school integration problems 68%, risky eating behaviors (binge-purge) 42%, risky eating behaviors (compensatory measurements) 21%, risky eating habits (restriction) 22%, and low family functionality 16%.

## Figures and Tables

**Table 1:** Distribution of individuals by religion.

Religion	%
Agnostic	0.48%
Atheist	0.73%
Catholic	49.88%
Christian	25.91%
None	21.79%
Jehovah’s Witness	1.21%

**Table 2:** Distribution by origin.

Origin	%
Not answer	8.23%
Rural	15.50%
Urban	76.27%

**Table 3:** Correlation matrix for different scales/sub-scales of the PANSI, BSSA-10, BQREM, and the APGAR instruments.

	BSSA-10 SP	BSSA-10 AE	BSSA-10 IP	BQREB BP	BQREB CM	BQREB R	APGAR	PANSI PNI	PANSI PSI
BSSA-10 SP	1.000								
BSSA-10 AE	0.122	1.000							
BSSA-10 IP	0.148	0.490	1.000						
BQREB BP	−0.067	−0.116	−0.094	1.000					
BQREB CM	−0.099	−0.055	−0.034	0.409	1.000				
BQREB R	0.012	0.041	0.016	0.288	0.207	1.000			
APGAR	0.049	−0.037	0.051	−0.021	−0.091	0.015	1.000		
PANSI PNI	0.027	0.024	0.012	−0.014	−0.015	0.032	−0.095	1.000	
PANSI PSI	−0.006	0.033	−0.061	−0.053	−0.020	0.021	−0.114	0.558	1.000

**Table 4:** Probabilities of developing suicide ideation as a consequnce of different factors from instruments BSSA-10, BQREB, and APGAR.

		PANSI-PNI			PANSI-SI		
	**RANGE**	**(8,19)**	**(20,29)**	**(30,40)**	**(6,14)**	**(15,22)**	**(23,30)**
**BSSA-10 SPA**	(3,8)	0.04	0.15	0.06	0.02	0.04	0.08
	(9,13)	0.06	0.20	0.11	0.04	0.07	0.15
	(14,18)	0.04	0.11	0.06	0.04	0.05	0.13
**BSSA-10 AE**	(2,5)	0.02	0.06	0.03	0.01	0.02	0.05
	(6,9)	0.04	0.11	0.07	0.01	0.04	0.09
	(10,12)	0.05	0.15	0.08	0.01	0.06	0.14
**BSSA-10 IP**	(5,13)	0.08	0.27	0.16	0.02	0.13	0.28
	(14,21)	0.06	0.16	0.09	0.02	0.06	0.08
	(22,30)	0.01	0.01	0.01	0.00	0.00	0.01
**BQREB BP**	(4,8)	0.04	0.12	0.06	0.02	0.04	0.07
	(9,12)	0.05	0.16	0.09	0.01	0.05	0.13
	(13,16)	0.03	0.11	0.07	0.02	0.04	0.12
**BQREB CM**	(3,6)	0.01	0.06	0.02	0.01	0.02	0.04
	(7,9)	0.03	0.09	0.04	0.01	0.04	0.09
	(10,12)	0.04	0.11	0.05	0.01	0.05	0.11
**BQREB R**	(3,6)	0.01	0.06	0.03	0.01	0.02	0.04
	(7,9)	0.03	0.08	0.05	0.01	0.05	0.09
	(10,12)	0.04	0.10	0.08	0.01	0.06	0.12
**APGAR**	(0,3)	0.01	0.03	0.02	0.00	0.01	0.02
	(4,7)	0.02	0.07	0.03	0.00	0.02	0.05
	(8,10)	0.02	0.12	0.07	0.02	0.05	0.0
